# A novel framework for the evaluation of coastal protection schemes through integration of numerical modelling and artificial intelligence into the *Sand Engine App*

**DOI:** 10.1038/s41598-023-35801-5

**Published:** 2023-05-27

**Authors:** Pavitra Kumar, Nicoletta Leonardi

**Affiliations:** Department of Geography and Planning, School of Environmental Sciences, University of Liverpool, Chatham Street, Liverpool, L69 7ZT UK

**Keywords:** Environmental sciences, Natural hazards

## Abstract

There is growing interest in the adoption of Engineering with Nature or Nature Based Solutions for coastal protection including large mega-nourishment interventions. However, there are still many unknowns on the variables and design features influencing their functionalities. There are also challenges in the optimization of coastal modelling outputs or information usage in support of decision-making. In this study, more than five hundred numerical simulations with different sandengine designs and different locations along Morecambe Bay (UK) were conducted in Delft3D. Twelve Artificial Neural Networking ensemble models structures were trained on the simulated data to predict the influence of different sand engines on water depth, wave height and sediment transports with good performance. The ensemble models were then packed into a *Sand Engine App* developed in MATLAB and designed to calculate the impact of different sand engine features on the above variables based on users’ inputs of sandengine designs.

## Introduction

Global sea level rise (SLR) and changes in extreme storms in connection to climate change pose a significant threat to anthropogenic activities along the coast. By 2300, the global sea level is expected to rise by at least 0.3 m and up to 16 m in the worst-case scenario^1^. Currently, more than 600 million people live along the coastline and face the consequences of coastal erosion^2^ and flooding^3^. These issues are being exacerbated by growing urban development and increasing population^4^. Studies have also shown that nearly one-quarter of global beaches are experiencing erosion^4,5^.

Given the ever-increasing coastal risks, and their impacts on human life and the economy, there have been growing efforts to look for efficient and cost-effective coastal protection methods. Traditional hard coastal defences such as breakwaters and seawalls have been successful but can be extremely expensive, have high maintenance costs and their adaptation to climate change is becoming economically unviable^6^. Working with Natural processes or Nature-Based solutions for coastal protection including mega-nourishment interventions or wetlands restoration can offer a more economically viable alternative and in addition to their coastal protection services, have the potential to support Net Zero-Carbon emissions, biodiversity, and local amenities^7^.

Mega-nourishment interventions are frequently referred to as sand engines and are very large, localized beach nourishments supporting safety against flooding as well as preventing coastal erosion in low-lying areas^8^. In particular, sand engines can attenuate wave energy and feed sediments to nearby coastlines sections over a time scale of the order of decades which is significantly longer than the one expected for traditional beach nourishment and as such, they offer reduced maintenance works over the long term^9,10^ because natural forces (wind, wave, and tides) distribute sediments along the coastline for several years. The first sand engine was built off the coast of South Holland in The Netherlands in 2011 and is more traditionally referred to as *Sand Motor* (https://dezandmotor.nl). The *Sand Motor* was built with 21.5 Mm^3^ of sand, having initially the shape of a hook with an area of 128 ha, and it is currently stretching more than 2.4 km along the coastline and 1 km off the coast^8^, thanks to the redistribution of sediments by natural drivers. This intervention was designed to last for at least 20 years^11^ and observations over the last five years suggest a longer lifetime than the initial design period^12^.

Although the shape of the sand motor has changed drastically since 2011, these changes are within the calculated limits. Simulated results^13,14^ and ground measurement^15^ showed that tidal forces spread a significant amount of sediments along the coastline. Four years (2011 to 2015) measurement revealed that despite all the sand movement, 95% of the deposited sand was still present at the location, suggesting a lifetime of more than 20 years^12^.

Sand Engines are an example of a paradigm shift in coastal management, consisting in moving away from the idea of “fighting” natural forces towards the idea of utilizing them for coastal protection^8^. Overall, in consideration of reduced maintenance costs and the considerable co-benefits that they offer, Nature-Based solutions including sand engines can represent a viable alternative for coastal protection^16^. However, when evaluating the effects of a Sand Engine on the surrounding areas depending on both Sand Engine features and site characteristics, there are still huge uncertainties about the functioning of the intervention in terms of energy dissipation as well as the influence of the sand engines on the overall hydrodynamics. Existing research suggests the need to critically study the behaviour of different sand engine designs^4^. Of course, practically implementing such massive interventions without prior know-how is not feasible. Laboratory, as well as numerical modelling, can be used for preliminary investigations and these rely on extensive resource usage. Within this context, Artificial Intelligence (AI) offers an interesting set of complementary tools which remain relatively unexplored within the coastal science field. It can help slim down resources usage through the development of algorithms solely focusing on a pre-defined set of variables, optimization of the usability of different modelling results within a separate external framework. Thus easing the implementation of such algorithms into light users-customizable applications which do not require prior knowledge on modelling.

This study focuses on the development of an operational framework for the evaluation of variations in wave height, water depth and sediment transport for different sand-engines configurations through the embedding of numerical modelling, performed on Delft3D, into an Artificial Neural Network (ANN). Followed by the development of a standalone Sand Engine App for results communication and prediction of sand engines effectiveness based on users’ inputs. Ensemble modelling is preferred to model these complex variations, as it has been successfully applied to model other complex relations such as coastal storm erosion prediction^17^, air pollution^18^ and its health risk prediction^19^, streamflow^20^ and water level prediction^21^, and groundwater quality modelling^22,23^. Specifically, 12 ensembles consisting of 96 different Recurrent Neural Network (RNN) and Feed Forward Neural Network (FFNN) models, is fed with data obtained from 552 simulations representing different sand-engines designs at 23 locations along the coastline of Morecambe Bay (UK). The simulated sand engines have different radii and heights and are tested with different wave conditions. Results from the ANN are then fed into a standalone application where users can input their sand engine features and obtain results about its effectiveness through the running of the ANN algorithms.

## Methods and data

### Methods overview

The hydrodynamics and sediment transport in Morecambe Bay were simulated using Delft3D and the outputs from the numerical models were then fed into the ANN models. Delft3D solves the 3-D Navier–Stokes equations for incompressible free-surface flow under the shallow water approximation for unsteady, incompressible, turbulent flow. The module Delft3D-WAVE was used to simulate wave generation, propagation, and nonlinear wave-wave interactions. Time series of depth-averaged velocity, water depth, significant wave height and sediment transport were extracted from several observation points located inside and outside the sand engines (at a buffer zone half of the radius of the sand engine) and fed into the ANN. Specifically, we used an ensemble of eight models structures: 4 Recurrent Neural Network (RNN) (i.e., 2 Elman Neural Network (ENN) models, 2 Layer Recurrent Neural Network (LRNN) models) and 4 Feed Forward Neural Network (i.e., 2 Cascade-Forward Neural Network (CFNN) models and 2 Feed-Forward Neural Network (FFNN) models). Each model structure was trained twelve times to predict twelve different sets of two variables (e.g. Table 1), leading to a total of 96 ANN models. Feeding results and training the ANN allowed the development of fast algorithms predicting mean and maximum variations in wave height, water depth and sediment transport for observation points within and outside the Sand Engine. Specifically, each of the eight model structures is developed into a model predicting maximum and mean water depth, significant wave height and suspended sediment transport, before and after the sand engine implementation, inside and outside the sand engine. ANN is then used as the basis for the *Sand Engine App* where Users can enter Sand Engine and coastline specifics (e.g., radius of the Sand Engine; wave height at the boundary; coastline inclination) and obtain results in terms of variations in wave height, water depth and sediment transport before and after the implementation of the Sand Engine.

**Table 1:** Performance of all ensemble model testing.

Model	Before sand engine			After sand engine		
	Test regression	Test MAE	Mean STD	Test regression	Test MAE	Mean STD
Mean						
Water depth (IN) (m)	0.9999	0.0055	0.0217	0.9950	0.0737	0.0710
Water depth (OUT) (m)	0.9999	0.0028	0.0108	0.9999	0.0090	0.0237
Wave height (IN) (cm)	0.9999	0.0610	0.3008	0.9995	0.3327	0.4901
Wave height (OUT) (cm)	0.9999	0.0145	0.2885	0.9999	0.0463	0.2884
Sediment transport (IN) (cm^3^/s/cm)	0.9999	0.0081	0.1900	0.9975	0.0255	0.2618
Sediment transport (OUT) (cm^3^/s/cm)	0.9999	0.0060	0.0230	0.9998	0.0191	0.0483
Maximum						
Water depth (IN) (m)	0.9419	0.7573	0.8610	0.9362	0.7918	0.7312
Water depth (OUT) (m)	0.9221	0.7042	0.9092	0.9261	0.6970	0.8997
Wave height (IN) (cm)	0.9388	3.3494	4.2492	0.8902	3.4662	3.2525
Wave height (OUT) (cm)	0.9769	2.5897	3.9645	0.9721	3.0452	3.8127
Sediment transport (IN) (cm^3^/s/cm)	0.9011	5.3613	7.4474	0.8728	4.6154	5.2015
Sediment transport (OUT) (cm^3^/s/cm)	0.9604	12.5810	15.7226	0.8804	10.2711	7.7354

### Numerical simulations

**Figure 1:**
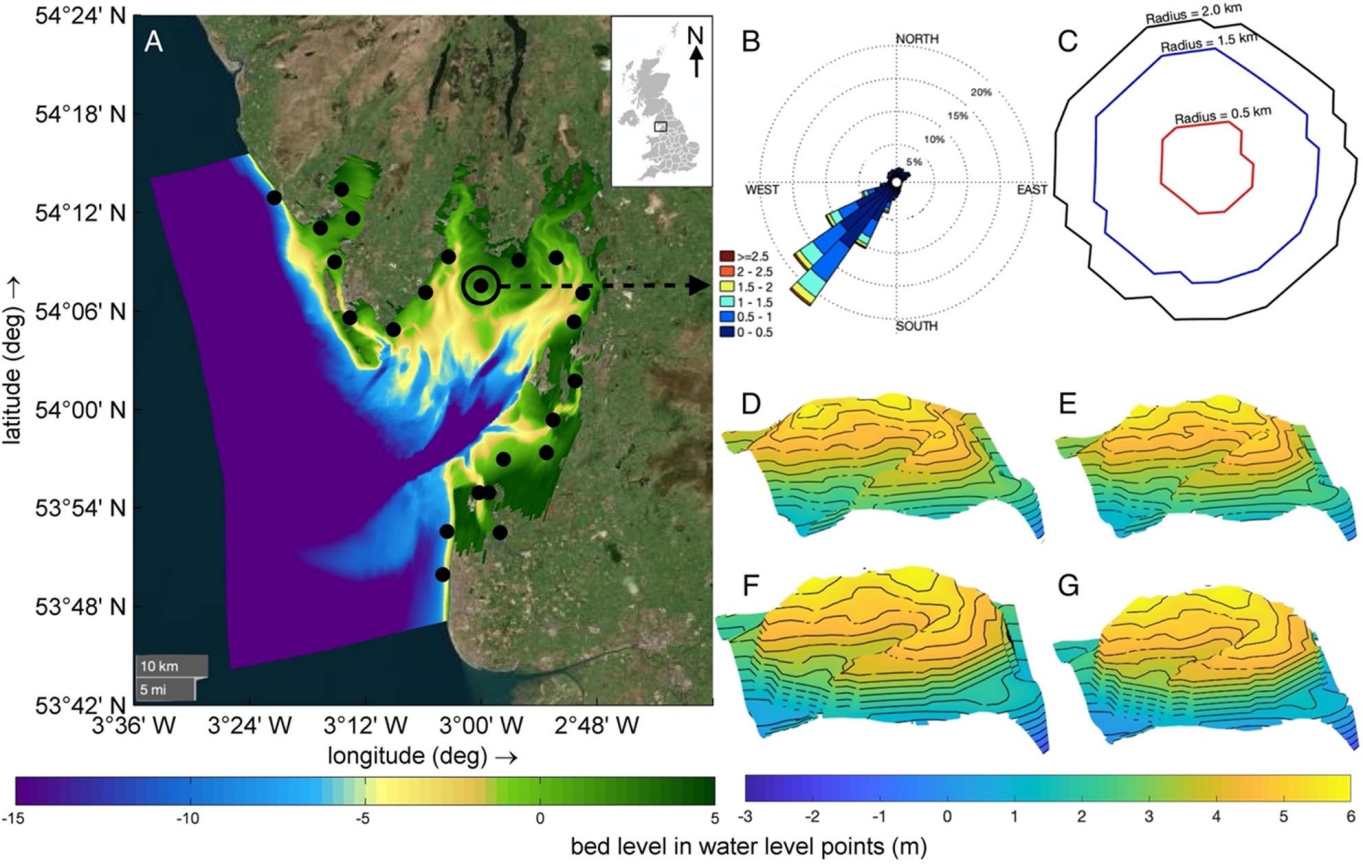
(**A**) Morecambe Bay model domain with the model bathymetry (colorbar located at the figure bottom). Locations with a sand engine are marked with black dots. (**B**) Wave rose presenting the wave climate in the area, the direction indicates waves provenance. Data are from the Northwest Regional Coastal Monitoring Programme (Data Copyright: Sefton Council. (**C**–**G**); (**C**) Examples of sand engines configuration: planar views for 0.5, 1.5 and 2 km diameter; Bathymetry of a sand engine of height (**D**) 0.5 m, (**E**) 1 m, (**F**) 2 m, (**G**) 3 m.

Morecambe Bay (Fig. 1A) is a large embayment, opening into the Irish sea, located in North-West of England. Most of its shoreline is covered in fine sand^24^. The bay experiences spring tidal waves with amplitude ranging up to 10 m. Fetch length for wind waves is constrained by Ireland and Isle of Man and sprints at the bay mouth. The significant wave height at the mouth of the bay can reach up to 2 m for about 10% of the year and for the remaining duration of the year significant wave height remains around 0.5 m^24^ (Fig. 1B). The hydrodynamics and sediment transport of Morecambe Bay was simulated on Delft3D. The model grid size varies from around 120 × 130 m onshore to around 1000 × 300 m offshore. The bathymetry used in the model was downloaded from EDINA Marine Digimap download service (https://digimap.edina.ac.uk/roam/download/marine). DTM data from LiDAR surveys at 2 m resolution were then used for areas covering the shoreline and were downloaded from the UK Environment Agency’s LiDAR data archive (https://environment.data.gov.uk/DefraDataDownload/?Mode=survey). The model boundary is forced with ten tidal harmonics (M2, S2, N2, K2, K1, O1, P1, Q1, S1, M4) interpolated across the two boundary extremes and derived from the global tidal model GOT-e 4.10c^25,26^. The module Delft3D-WAVE was used to simulate wave generation, propagation, and nonlinear wave-wave interactions. Within this module, bottom dissipation, whitecapping, and depth-induced breaking are fully accounted for in a dissipation term^27^. The model was calibrated using OpenDA^28–31^ and through a comparison of the simulated water level values with values at the Heysham tidal station (https://ntslf.org/data/uk-network-real-time). OpenDA interfaces with Delft3D and uses a derivative-free algorithm (DUD or doesn't use derivative)^32^, an algorithm for non-linear least squares minimization, to minimize a quadratic cost function based on differences between observed and model water levels through changing of roughness coefficient, water depth and boundary conditions. Successive iterations of the numerical simulation were repeated until the convergence criteria were reached. The accuracy was evaluated using the Brier Skill Score^33^ defined as:

1 $$BSS = \frac{\alpha - \beta - \gamma - \varepsilon }{{1 + \varepsilon }}$$

where $\alpha = r_{XY}^{2}$, $\beta = \left( {r_{XY} - \frac{{\sigma_{Y} }}{{\sigma_{X} }}} \right)^{2}$, $\gamma = \left( {\frac{\left\langle Y \right\rangle - \left\langle X \right\rangle }{{\sigma_{X} }}} \right)^{2}$, $\varepsilon = \left( {\frac{\left\langle X \right\rangle }{{\sigma_{X} }}} \right)^{2}$ for which *r* is the correlation coefficient, σ is the standard deviation (STD), ε is a normalization term, and X and Y are observed and modelled values. The model was calibrated from January 5th to February 20th, 2018^34^. The Brier Skill score in this case was 0.99. The model was then run for 5 days, with a time step of 1 min to encompass 10 tidal cycles. Non-Cohesive sediment type with a specific density of 2650 kg/m^3^ and dry bed density as 1600 kg/m^3^ was used for simulating the sediment transportation. Depth averaged (2DH) advection–diffusion equation is solved for suspended sediment load calculation^35,36^. Van Rijn^37^ separated bedload from suspended load based on a reference height (0.05 m for this case), above which is considered as suspended load transport and below which is considered as bedload. The depth-averaged equilibrium concentration, solved using expressions provided by Van Rijn^38^, is used for the calculation of sediment exchange between the bed and water column, which includes computation of velocity profile and vertical concentration profile. Near-bed reference concentration (C_a_), computed by Eq. (2), is required to compute the vertical sediment concentration profile.

2 $$C_{a} = 0.015\left( {\frac{{D_{50} }}{a}} \right)\frac{{\left( {\frac{{\tau^{\prime}_{b,cw} - \tau_{b,cr} }}{{\tau_{b,cr} }}} \right)^{1.5} }}{{D_{*}^{0.3} }}$$

where: $\tau_{b,cr}$ is the critical bed shear stress, $\tau^{\prime}_{b,cw}$ is grain related bed shear stress due to current and waves, *D*_50_ is median sediment diameter (120 μm, in this case), a is Van Rijn’s reference height and $D_{*}$ is non-dimensional grain size. The depth-integrated suspended load transport is calculated by Eq. (3).

3 $$\overrightarrow {{q_{s} }} = \overrightarrow {U} ch$$

where: $\overrightarrow {{q_{s} }}$ is depth-integrated suspended sediment transport, $\overrightarrow {U}$ is depth-averaged velocity, *c* is depth-integrated sediment concentration and *h* is water depth.

Twenty-three circular Sand engine locations along the coastline of Morecambe Bay were tested with sand engines having a different radii (0.5 km, 1.5 km, and 2 km) (Fig. 1C) and different height (0.5 m, 1 m, 2 m, and 3 m) (Fig. 1D–G). Each sand engine was simulated individually under varying boundary conditions with wave heights of 0.5 m and 1 m imposed at the south-west of the domain, where the water depth varies from about 24 to 30 m along the boundary, The wave direction was orthogonal to the boundary following the wave rose for the site (Fig. 1B). Water level boundary condition was used at sea boundary (south-west of the domain) where waves and tides were imposed. Neumann boundary condition was used for the lateral boundaries.

Each sand engine has its inner and outter observation points distributed around as shown in Fig. S1. For each sand engine simulation, an identical model was run without the sand engine to compare the effect of the latter on wave height, water depth and sediment transport. A total of 552 sand engine models were simulated encompassing all the above-mentioned configurations. The time series of different variables were recorded at the observation points inside the sand engine and outside sand engine. Mean and maximum for each time series were used to train ensemble models to predict the effect of sand engines on the mean and maximum of the three variables (water depth, significant wave height, and sediment transport) both inside and outside the sand engines.

### Ensemble modeling

**Figure 2:**
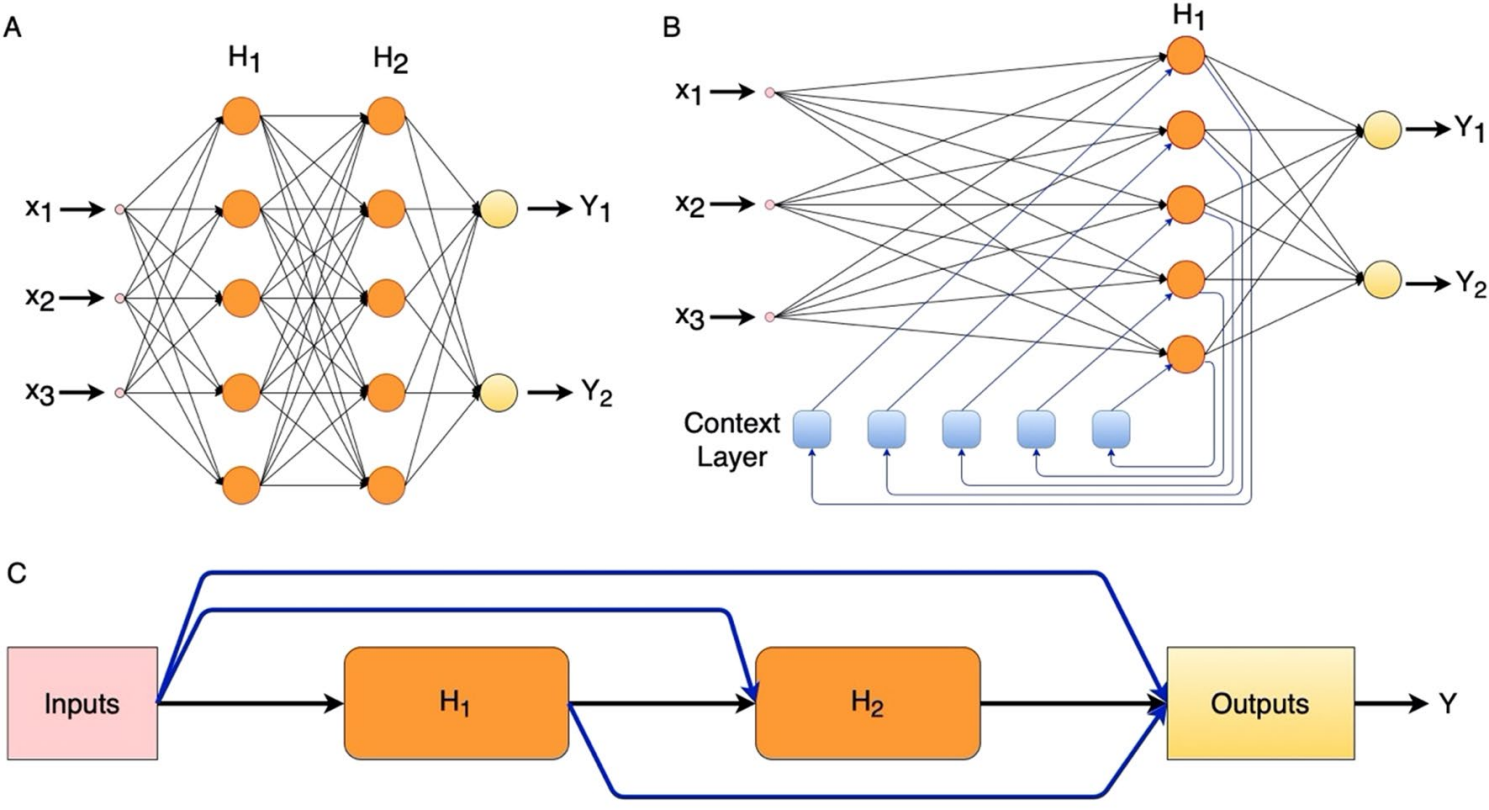
Basic structure of (**A**) FFNN and (**B**) ENN (**C**) CFNN (x_1_, x_2_ and x_3_ are inputs to the model, H_1_ and H_2_ are the hidden layers, and Y, Y_1_ and Y_2_ are the outputs of the models).

ANN is a black-box model^39,40^, the internal structure of which is similar to the human brain^41–43^. The function of ANN is to provide prediction values based on the historical data on which it is trained. Training allows ANN models to learn the relationship between input and output variables^39,44,45^ and these relationships are then used for future predictions. ANN models can also learn non-linear relationships between different variables^46,47^. Figure 2A represents a simplified version of the structure of the Feed Forward Neural Network (FFNN) which was used for this work. The Internal structure of FFNN consists of an Input layer, hidden layers, and an output layer with multiple nodes in each layer. The number of nodes in the input layer depends on the number of inputs to the model^48^. The number of hidden layers and corresponding nodes in them depends on the level of complexity required to model the relationship between the variables and more complex relationships usually require more number of nodes and hidden layers. The number of nodes in the output layers depends instead on the number of prediction outputs the model is providing^48^. All the nodes of one layer have a connection with those of the next layer^49^ and data are received, processed, and transferred through these nodes^50^. The data received at the node are processed (multiplied with the respective connection weights followed by adding the biases [Eq. (4)]^51^ and transferred using a transfer function. Out of several transfer functions, two of them are used in this model for each node: log-sigmoid transfer function [Eq. (5)] for all nodes of hidden layers^52^ and linear transfer function for nodes in output layer. Models were trained using two training functions: Levenberg–Marquardt *(trainlm)* for ENN and LRNN and Bayesian Regularization *(trainbr)* for FFNN and CFNN models. While training the model, weights and biases are updated at the nodes using the back-propagation algorithm^53^, which propagates the error produced by the model backwards from the output layer to input layer through hidden layers updating accordingly the weights and biases in every iteration of training, aimed to reduce the final error^54,55^.

4 $$\gamma =f\left(\sum \left({w}_{i}*{\phi }_{i}\right)+\beta \right)$$

5 $$f\left(A\right)=\frac{1}{1+{e}^{-A}}$$

where: $\gamma$ is the processed value calculated by multiplying weight w with the value $\phi$ from the nodes of previous layer and adding biases $\beta$ followed by transferring through a tan-sigmoid transfer function $f\left(A\right)$ (A is any function value).

The structural difference between FFNN and other models used in this study are additional layers (in ENN and LRNN) and connections (in CFNN). Figure 2B presents the basic structure of ENN with additional context layer and Fig. 2C represents the basic structure of CFNN with additional connections between input, hidden and output layers. The special feature of ENN is its context layer, which stores a copy of the information to be provided to the hidden layers in the subsequent calculation steps^56^, thus serving as a memory to the ENN as it holds a copy of activations of previous time step^57,58^. Each hidden layer of ENN has its context layer with the number of nodes equal to the number of nodes in the corresponding hidden layer. The ENN model is chosen for this study because of its memorizing capability, through its additional context layer, which provides it with the characteristic of being time-varying and having global stability^59,60^. Also, ENN is well known for its capability of dynamic modelling^61^. LRNN has the structure similar to ENN, thus, bearing all the advantages of ENN with additional forward propagation and backpropagation dynamic derivative function, which help in calculating derivatives using chain rule from input to output (in case of forward propagation) and from network’s performance back through the network (in case of backpropagation), thus helping in model learning. CFNN has similar structure to FFNN except for the additional connections between the layers which helps in exploring the dependency of target data upon input data^62^. Each layer in CFNN receives a direct connection from the input layer and all preceding layers. These connections help in accommodating non-linear relationships between input and target without eliminating their linear relationship^63^.

Data obtained from all 552 sand engine simulations were used to train the ensemble ANN models for predicting water depth, significant wave height and sediment transport with and without the presence of a sand engine. Specifically, the data extracted from one simulation are the mean and the maximum of each interested variable at all observation points inside and all observation points outside the sand engine. The network was developed with the idea of utilizing the least possible number of input variables whose knowledge does not require prior detailed modelling or data collection on the site. Therefore, in addition to the main sand engine features (height and radius of a sand engine) the following were selected as input variables at the nodes: wave height at the boundary for an overall indication of wave conditions at the site, the distance of the sand engine from the boundary for a broad indication of how far inside the embayment the sand engine is, angle of the coastline at the location of the sand engine and depth average velocity at the same site (without the presence of sand engine). Angle of coastline was calculated in 360-degree format clockwise with respect to the simulated sea boundary (which is at 164.6 degree from north). These input values were used to predict the mean and maximum values of water depth, significant wave height and sediment transport inside and outside the sand engine before and after sand engine’s presence. A total of 12 ensembles (each consisting of 8 models structures) were trained to predict 24 different output values [i.e., 2 types (mean and maximum) for 3 output variables for 2 locations (inside and outside) for 2 conditions (with and without sand engine)]. Each ensemble was trained to predict two sets of outputs i.e., output variables with and without the sand engine. The value of the output variables with a sand engine has some dependency on the corresponding value without the sand engine. For instance, the reduction in water depth due to the presence of a sand engine is dependent on the initial water depth at that location without a sand engine.

**Figure 3:**
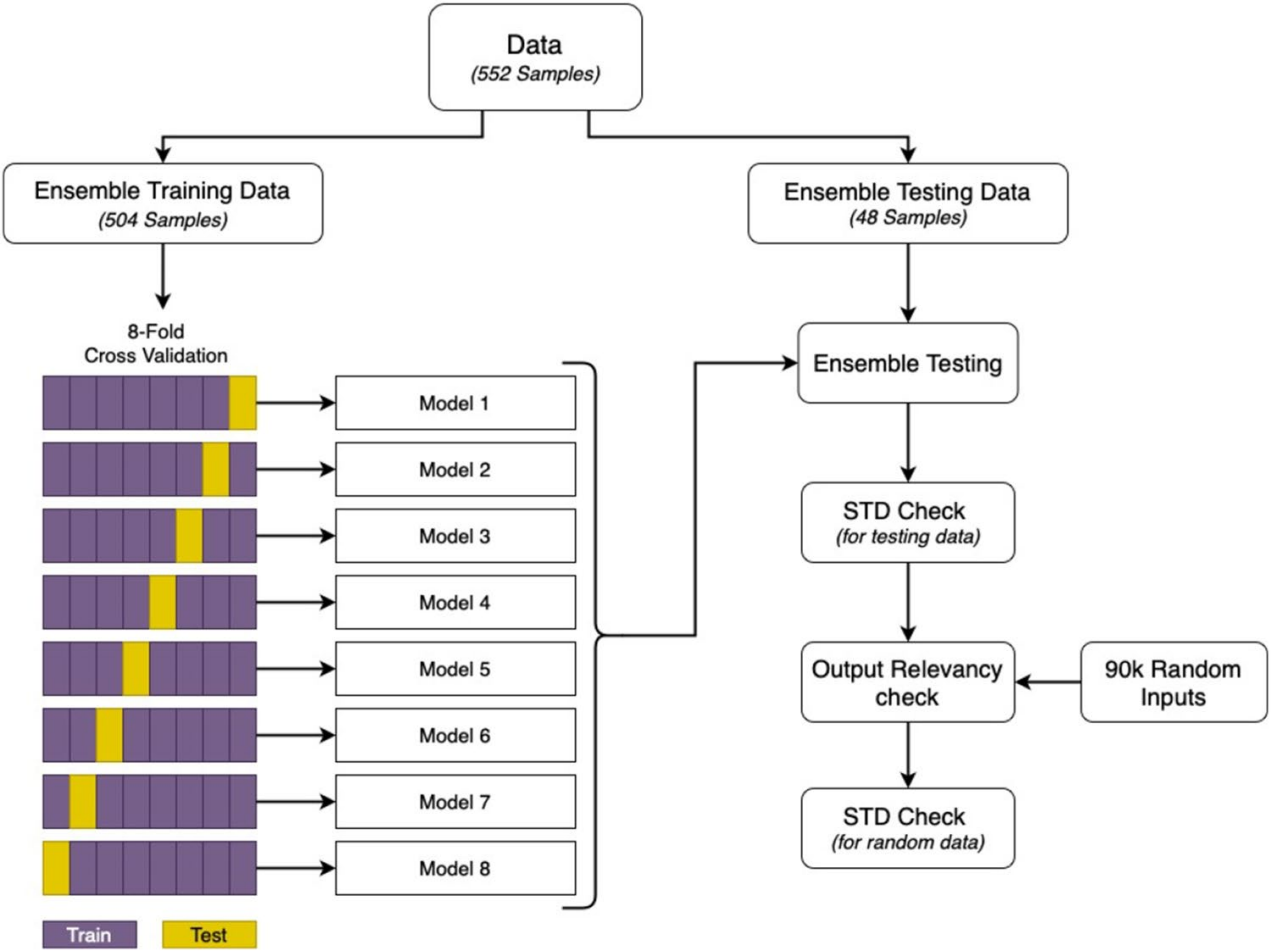
Ensemble training methodology.

Each ensemble was trained using eightfold cross-validation. Figure 3 represents the process of training and testing ensemble models. The available data (552 samples) were split into training and testing data with 504 and 48 samples each, respectively. The division is close to 90–10 split with numbers selected such that the training dataset could be further split into 8 equal bins for eightfold cross-validation. Following the procedure of k-fold cross-validation, 8 models (2 ENN, 2 LRNN, 2 CFNN and 2 FFNN) were trained on 7 bins and tested on 8th bin, provided the testing bin was different for all 8 models, as shown in Fig. 3. The trained ensemble was tested on the testing dataset (48 samples) separated earlier. The outcome of the ensemble was calculated as the median of outputs of all 8 models after eliminating the negative outputs (if any), as the interested output variables cannot have negative values. STD was calculated to get the deviation of output of each model from the median result of the ensemble. However, all models are trained on different training and testing datasets, thus, are liable to produce different outputs^64^. Apart from testing the ensemble models on the separated testing dataset, they were also tested for the output relevancy and STD on more than 90 k randomly generated inputs close to the training range. To generate these random inputs, all the 6 inputs were varied within the following range: height of sand engine was varied from 0.5 to 3 m with an interval of 0.5 m, radius of sand engine was varied from 0.5 to 2 km with an interval of 0.5 km, wave height was varied from 0.5 to 1 m with an interval of 0.25 m, distance was varied from 15 to 45 km with an interval of 5 km, angle was varied from 20 to 360° with an interval of 20° and average velocity was varied from 0.01 to 0.5 m/s with an interval of 0.05 m/s. All possible combination of these variation of 6 inputs were made which added to 90,720 random inputs. These 90 k random inputs were used to test the output relevancy of the ensemble model, where relevant output stands for non-negative output of the ensemble model. As either of the interested target variable cannot have negative values, hence, a negative prediction from the ensemble model is considered an irrelevant output.

## Results

**Figure 4:**
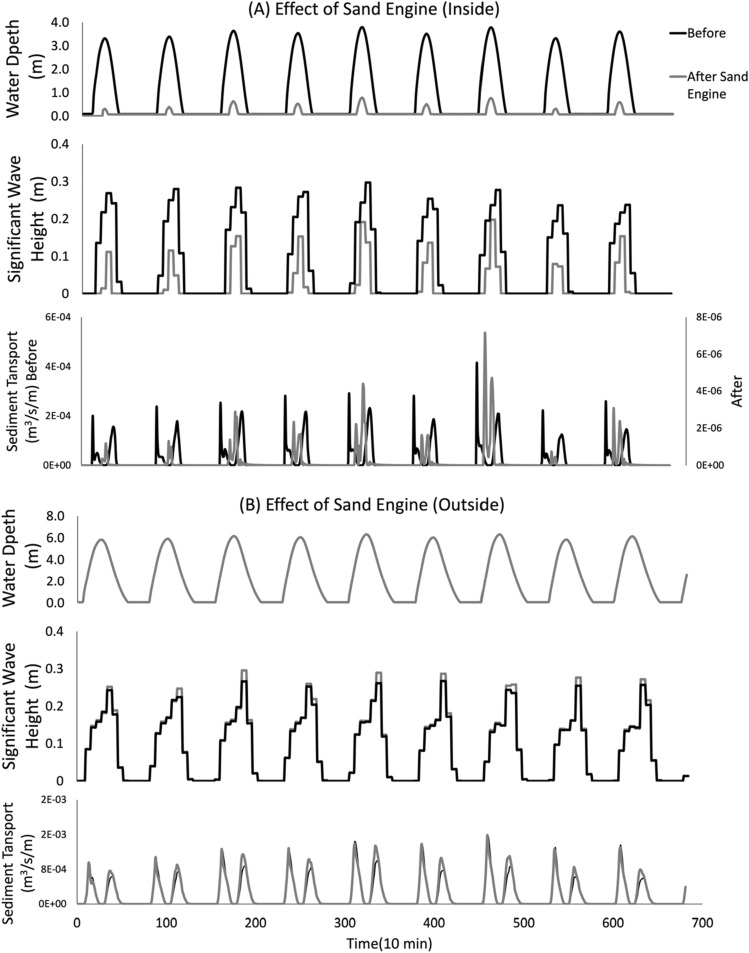
Example of effect of sand engine implementation.

Figure 4 provides an example of simulation results and illustrates the impact of one of the tested sand engines in terms of water depth, significant wave height and sediment transport. Results are presented for a sand engine having 3 m height and 1.5 km width. The location of the sand engine in analysis is indicted by the double circle in Fig. 1A. The significant wave height at the boundary for the simulation is 1 m. Subpanels A and B refer to values for a specific wet observation points inside and outside the sand engine.

The performance of all ensemble models on the testing dataset is presented in Table 1. These values represent how well the ensemble models performed in predicting a given variable given the following inputs: wave height at the boundary; distance of the sand engine from the boundary; angle of the coastline at the location of the sand engine; depth-average velocity at the same site (without the presence of sand engine). The predictive performance of ensemble models was measured based on three performance criteria: Regression [Eq. (6)], Mean Absolute Error (MAE) [Eq. (7)] and standard deviation (STD) [Eq. (8)]. Regression provides the statistical measure of how the predicted data fits with the target data, thus defining the generalizing capability of the model. However, regression criterion alone cannot define the accuracy of the model^65^. Thus, MAE was included in the performance criteria, which provides a measure of error in the predicted values. Regression is calculated as:

6 $$r=\frac{n\left(\sum xy\right)-\left(\sum x\right)\left(\sum y\right)}{\sqrt{\left[n\sum {x}^{2}-{\left(\sum x\right)}^{2}\right]\left[n\sum {y}^{2}-{\left(\sum y\right)}^{2}\right]}}$$

7 $$MAE=\frac{1}{n}\sum_{i=1}^{n}\left|x-y\right|$$

STD along median (for this study) is calculated as:

8 $$\sigma =\sqrt{\frac{\sum_{i=1}^{n}{\left({x}_{i}-m\right)}^{2}}{n}}$$

where: n is the number of data points, x is target value, y is predicted value, $\sigma$ is STD and m is median

All the models within the ensemble were trained several times by varying the hidden layers (2 and 3) and nodes within them (5 to 25) and the model providing least testing MAE was selected for further processes. The training and testing accuracy of all 96 models are presented in Table S1. Each ensemble model was trained to provide two outputs (before and after sand engine), hence the testing accuracy of the models is measured separately for both the outputs, as presented in Table 1. Models trained on mean data provide predictions in relation to mean output variables, while those trained on maximum data predict the maximum in the output variables. The models for maximum prediction are less accurate in comparison to models for mean prediction, the reason being the training data of mean is more uniform in comparison to that of maximum data. The mean absolute error for maximum water depth is about 0.8 m while the maximum water depth observed at the location of sand engines is about 20 m. Since the maximum wave height at the boundary of the Morecambe Bay is around 2.0 m, and in situ and for the location of the sand engines is around 1 m for those closer to the boundary and about 0.35 m for those farther away from the boundary, the maximum mean absolute error of about 3.5 cm is considered acceptable. Similarly, the maximum sediment transport simulated with sand engine was recorded around 200 cm^3^/s/cm and the mean absolute error is around 13 cm^3^/s/cm which is considered acceptable.

Table 2 presents the analysis of all ensemble models on 90 k random inputs. No. of irrelevant output column presents the number of inputs, out of 90 k inputs, at which corresponding ensemble model provided negative outputs. The ensemble model predicting sediment transport outside the sand engine is providing more STD and it has less accuracy in for testing data (Table 1), due to the bad training data.

**Table 2:** Analysis of ensemble models using 90 k random inputs.

Model	Before sand engine		After sand engine	
	No. of irrelevant outputs	Mean STD	No. of irrelevant outputs	Mean STD
Mean				
Water depth (IN) (m)	18	1.6035	78	1.7940
Water depth (OUT) (m)	42	1.8067	36	1.8656
Wave height (IN) (cm)	529	6.7085	270	8.2437
Wave height (OUT) (cm)	114	7.0349	123	7.0372
Sediment transport (IN) (cm^3^/s/cm)	45	0.7647	0	4.0602
Sediment transport (OUT) (cm^3^/s/cm)	68	1.3755	50	1.6399
Maximum				
Water depth (IN) (m)	0	3.6174	51	3.4053
Water depth (OUT) (m)	0	5.0449	0	5.1096
Wave height (IN) (cm)	80	16.9346	294	16.3706
Wave height (OUT) (cm)	0	27.4876	10	26.6457
Sediment transport (IN) (cm^3^/s/cm)	10	98.2375	5	53.2448
Sediment transport (OUT) (cm^3^/s/cm)	0	201.0789	42	35.0140

### Sand Engine App

**Figure 5:**
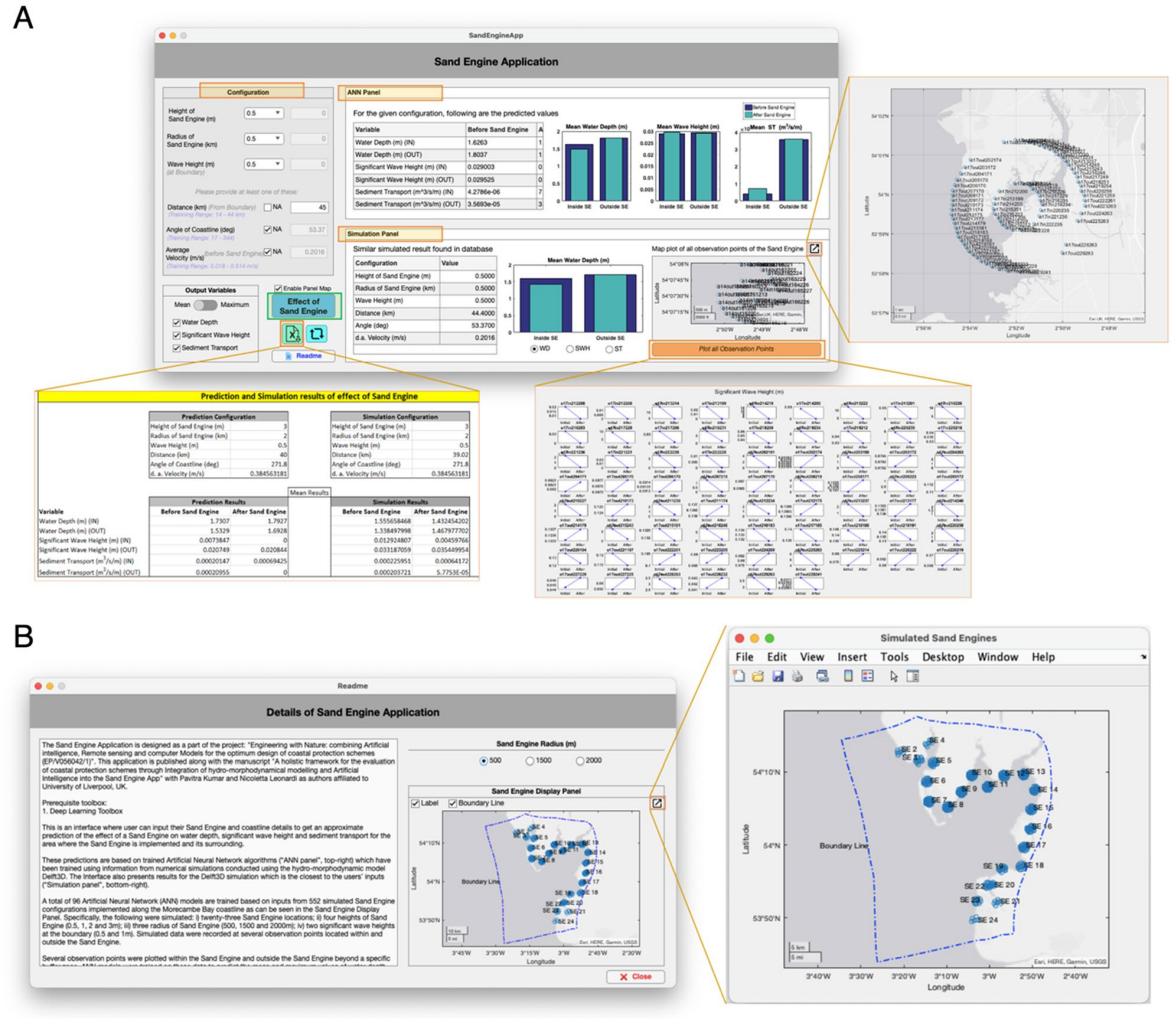
(**A**) Sand Engine Application; (**B**). Readme section of Sand Engine Application.

The trained ensemble models are packed into a MATLAB application [*Sand Engine App* (Fig. 5A)] for end users to receive outputs of the effect of a sand engine on the basis of their own inputs. This application runs on the MATLAB platform with one pre-requisite toolbox (Deep Learning Toolbox) available in the MATLAB add-ons (see SI, Video 1).

The *Sand Engine App* consists of an input panel (*Configuration Panel*) and two output panels (the *ANN Panel* and the *Simulation panel*). The App includes options for data export and a mapping visualization option. The *Sand Engine App* is available for download from the repository (https://github.com/pavitra979/SandEngine.git). The *Configuration Panel* consists of six input options: height of sand engine, radius of sand engine, wave height at the boundary, distance of the sand engine from the boundary, angle of the coastline at the location of sand engine, and average velocity at the location before sand engine. To obtain information on the effect of the sand engine, users are required to input at least four of the above input variables. Each input subpanel provides information about the range of data on which the ensembles models were trained on, in the form of drop-down list for first three inputs and in the form of a range of values for the last three inputs. The ANN models are more likely to provide accurate results if the input variables are close to their training ranges^66–68^.

The *Configuration Panel* includes an *Output Variables* option to select the variables to visualize in the results panels. It also includes a switch to select mean or maximum, which will display the analysis in relation to the mean or the maximum. The *ANN Panel* of the application displays prediction values in tables and plots. Each output variable has a separate plot consisting of four predicted values: before and after implementing a sand engine and inside and outside sand engines. For the ANN Panel, results are based on the ANN algorithms developed in this article as mentioned in the above sections.

For completeness, we have included a *Simulation Panel* displaying results from the numerical simulation which is the closest to the input parameters in the input section panel. While the *ANN Panel* displays results on the basis of ANN algorithms, the *Simulation Panel* is static and displays an example from a numerical case which is the closest to the user case. In addition to summary results for the impact of a sand engine, The *Simulation Panel* includes a map plot to display the location of sand engines and their inside and outside observation points inside Morecambe Bay, and a button to expand results for every observation point. Finally, the *Sand Engine Application* has a feature of exporting the predicted and simulated results into an excel format.

The location of all 23 sand engines simulated in this study can be visualised all at once in a map presented in readme section within the configuration panel (Fig. 5B). This section has radio buttons to plot sand engines of all radii (0.5 km, 1.5 km and 2 km) used in this study. The “Boundary line” (Fig. 5B) tick box option allows displaying the boundary line of the simulation domain along with all the 23 sand engine, thus providing better visualization of the location of the sand engines with respect to the simulation boundary. Additionally, it contains the description of different parts of sand engine app thus easing the usability of the app.

## Discussion

There are ever-growing concerns over the risks faced by coastal communities in the face of climate change and with the increasing urbanization of the coastline. At the same time, there has been a growing interest in the idea of Nature Based Solutions (or Working with Natural Processes) for coastal protection both in terms of flood risk management and coastal erosion management. This is in consideration of the increasing costs associated with hard-infrastructures maintenance as well as the co-benefits offered by such interventions including having the potential to support Net Zero target emissions and the creation of local amenities and recreational spaces. The potential of Nature Based Solutions has indeed been recognized in several regulatory frameworks. As an example, some of the UK strategies (https://assets.publishing.service.gov.uk/government/uploads/system/uploads/attachment_data/file/693158/25-year-environment-plan.pdf25) explicitly mentioned the importance of working with nature in support of flood risk reduction. At the same time, the UN called for action in terms of ecosystems restoration, by declaring 2021–2030 as the “decade on Ecosystem Restoration”. Among the others, coastal habitats have been recognized as some of the areas with the lowest standards in conservation, ecological and environmental terms^69,70^.

However, the implementation of Nature-Based Solutions is not straightforward. Sánchez-Arcilla, Cáceres^70^ summarize some of the challenges in relation to their implementation by highlighting a set of technical, Financial and Governance barriers. Some of the barriers are interlinked and related to limited engineering expertise when dealing with a multitude of ecological and environmental factors. Such limitations, frequently lead stakeholders or coastal managers as well as the overall public opinion to lend towards the adoption of more traditional hard defences. For instance, Schuerch, Mossman^71^ focus on Managed realignment options to highlight how these interventions can face mistrust by local communities not only because these interventions might require giving back to the sea previously utilized land but also because of the significant uncertainties in the use of such management actions.

A Sand-engine is one example of Nature Based solution having the potential to be economically viable when large volumes of the appropriate sediments are available for its implementation. There have been successful examples of mega nourishments, notably the Sand Motor in the Netherlands, and smaller-scale beach nourishment operations have been a traditional practice around the world for many years. However, in spite of the numerous studies on the topic, there are still many uncertainties on the impact of such interventions depending on their location, environmental conditions and geometrical features of both intervention and coastline. Furthermore, there is currently no consistent way of communicating and integrating results from different numerical or field experiments into a unique framework and in support of decision-making.

Within this context, this manuscript has proposed a novel framework presenting the initial methodological steps for the creation of novel tools aimed at supporting decision-making, gathering information from multiple sources to feed into a unique tool and, providing information on the effectiveness of different sand engines options on the basis of users’ inputs.

Specifically, the proposed framework integrates numerical modelling with Artificial Neural Networking into a *Sand Engine App* to provide information on significant wave height, water depth and sediment transport before and after the implementation of a Sand Engine. The example proposed in this manuscript focuses on Morecombe Bay. The proposed framework does not require detailed user inputs and it is designed to provide the mean and maximum values of all variables at locations inside and outside the sand engine. As a reference, the framework also provides complete information about the simulated results having a configuration which best matches users' inputs. The Simulation panel displays the map with plotted observation points both at inside and outside locations. Also, the mean and maximum effect of a sand engine can be viewed at all observation points. The sand engines simulated for this study can be viewed all at once in the readme section of the App, which also displays the boundary line of simulation domain for better understanding of the location of each sand engine with respect to the boundary and with respect to the coastline. Readme section has the feature of plotting sand engine of all the three radii simulated in this study. The displayed map can be enlarged for better visualisation.

The obvious limitation of this study is that the location and training of data are limited to one study site. The prediction model is trained on a limited set of simulation data, as mentioned in the framework, hence, it is more likely to provide better results when users' inputs are close to this range. The next steps for this framework will be to increase the amount of information fed into the ANN model to include a wider set of environmental conditions as well as different study sites. This will require formatting the input datasets in such a way that they can feed into the existing Network, but it doesn’t present stringent limitations in terms of data sources that could potentially be fed into it. Another limitation is the static nature of the simulation used in this study. Sand Engines are expected to change their shape and bathymetry over time when waves and tides are imposed, as observed in the sand motor in The Netherlands^9,72,73^. The latter was originally hook shaped stretching more than 2.4 km along the coastline and 1 km off the coast^8,74^ and has retreated 150 m towards the coastline and extended 1200 m alongshore within 18 months since the establishment^9^. The sand motor peninsula lost around 1.8 million m^3^ of sand within the first 18 months, which is 10% of nourished volume^9,15^. In this study, we did not focus on the morphological evolution of Sand Engines but rather on the initial effect of sand engines on a set of hydrodynamic and sediment transport variables inside and outside the sand engine.

We expect that allowing the sand engine to morphologically evolve over time will lead to varying effect of water depth, wave height and sediment transport both inside and outside the sand engine. In this sense the output variables determining the efficacy of the sand engine will change over time. Predicting non-stationary time-series for these variables will likely require complex RNN models such as Long Short-Term Memory (LSTM) model to handle multiple time series^75,76^. To predict the varying effect over time, the model has to predict complete time series based on different feature inputs (sand engine and wave configurations), which requires additional connection modifications and arrangements of LSTM cells, based on use case, as done by Wang, Fan^77^. Wang, Fan^77^ arranged 501 LSTM cells in parallel to predict complete time series, based on different feature inputs, where each cell was predicting a time step of the series. In their case, the connection of the LSTM cells was modified such that each cell was predicting based on predictions of previous 16 cells. Studying the varying effect of evolving sand engine on water depth, wave height and sediment transport and developing complex LSTM models for predicting the same is recommended for future research.

The *Sand Engine App* is uploaded in a public repository with its link given at the beginning of this article which can be downloaded and directly installed in MATLAB. All the ENN models and required files are integrated into the installation file of the framework, hence it does not require any network connection for usage. The framework has the advantage of running all 12 prediction models at once and presents the results in a meaningful manner along with the closest simulation results. However, it requires MATLAB to be pre-installed with one requisite toolbox: the deep learning toolbox, which can be easily searched and downloaded from the Add-ons option in MATLAB. A demo video and a description file, explaining the installation and usage, are also uploaded along with the framework in the repository. It is recommended to view those files before using the App.

## Conclusion

This article presents a novel framework supporting the choice of coastal protection schemes through the synthesis of numerical modelling outputs into an Artificial Neural Networking model whose computational efficiency allows the creation of a standalone computer application (*Sand Engine App*) illustrating the effectiveness of different users’ defined sandengines. The article illustrates the potential of synergies and complementarity between numerical modelling and Artificial Intelligence techniques. A total of 552 simulations using Delft3D were conducted with different sand engine configurations to look at their influence on the water depth, significant wave height and sediment transport at Morecambe Bay (UK). Simulation data were recorded at observation points placed inside the sand engine and outside the sand engine at a buffer zone equivalent to half of the radius of the sand engine. An ensemble of recurrent neural networks (ENN and LRNN) and feed forward neural network (FFNN and CFNN) was trained for the prediction of mean and maximum variable changes due to sand engine presence. Specifically, 12 ensemble models were trained to predict 24 different variables: [i.e., mean and maximum of 3 output variables (significant wave height, water depth, sediment transport) at 2 locations (inside and outside) and for 2 conditions (with and without sand engine)]. Ensemble models provided good accuracy with majority of testing regression greater than 0.90. Results from all ensemble models were packed into the *Sand Engine App* which is available for download (https://github.com/pavitra979/SandEngine.git).

## Supplementary Information

Supplementary Table S1.

Supplementary Figure S1.

Supplementary Video 1.

## Data Availability

Bathymetry data have been retrieved from EDINA Marine Digimap (http://digimap.edina.ac.uk/) and UK Environment Agency’s LiDAR data archive (https://environment.data.gov.uk/DefraDataDownload/?Mode=survey) which are gratefully acknowledged. The Data drive models have been developed using the MATLAB libraries from the Deep Learning toolbox [e.g. feedforwardnet(), elmannet(), cascadeforwardnet(), layrecnet(), and train()]. The Sand Engine App (Fig. S1) is available to download as part of the Supplementary material. This requires MATLAB and the MATLAB Deep Learning toolbox to run. A video (Video 1) in support of the installation is also provided in the Supplementary material. The App is available here: https://github.com/pavitra979/SandEngine.git.

## Code availability

Name of the Software: SandEngineApp. Developer: Pavitra Kumar and Nicoletta Leonardi. Contact Information: pavitra.kumar@liverpool.ac.uk. Year First Available: 2022. Platform: MATLAB. Required Library: Deep Learning Toolbox. Cost: Free. Software Availability: https://github.com/pavitra979/SandEngine.git. Program Size: 4 MB.
