# Supplementary material for: A novel framework for the evaluation of coastal protection schemes through integration of numerical modelling and artificial intelligence into the Sand Engine App
Source: Sci Rep. 2023 May 27;13:8610. doi: 10.1038/s41598-023-35801-5 (PMC10224936; doi:10.1038/s41598-023-35801-5)
Supplement: Supplementary file 1 — Supplementary Table S1. [file 41598_2023_35801_MOESM1_ESM.docx]

**Table S1.** Accuracy of all models in all Ensembles

| **Model** | | **Ensemble Model** | **Before Sand Motor** | | | **After Sand Motor** | | |
| --- | --- | --- | --- | --- | --- | --- | --- | --- |
|  |  |  | **Train Regression** | **Test Regression** | **Test**  **MAE** | **Train Regression** | **Test Regression** | **Test**  **MAE** |
|  | **Mean** | | | | | | | |
| Water Depth (IN)  (m) | | ENN1 | 0.9999 | 0.9999 | 0.0165 | 0.9999 | 0.9991 | 0.0336 |
|  |  | ENN2 | 0.9999 | 0.9999 | 0.0139 | 0.9998 | 0.9992 | 0.0617 |
|  |  | LRNN1 | 0.9999 | 0.9997 | 0.0218 | 0.9999 | 0.9990 | 0.0385 |
|  |  | LRNN2 | 0.9999 | 0.9999 | 0.0168 | 0.9999 | 0.9996 | 0.0369 |
|  |  | CFNN1 | 0.9999 | 0.9999 | 0.0225 | 0.9995 | 0.9993 | 0.0497 |
|  |  | CFNN2 | 0.9999 | 0.9999 | 0.0138 | 0.9999 | 0.9995 | 0.0259 |
|  |  | FFNN1 | 0.9999 | 0.9999 | 0.0052 | 0.9999 | 0.9992 | 0.0339 |
|  |  | FFNN2 | 0.9999 | 0.9999 | 0.0076 | 0.9999 | 0.9985 | 0.0504 |
| Water Depth (OUT)  (m) | | ENN1 | 0.9999 | 0.9999 | 0.0142 | 0.9999 | 0.9999 | 0.0377 |
|  |  | ENN2 | 0.9999 | 0.9999 | 0.0105 | 0.9999 | 0.9919 | 0.0687 |
|  |  | LRNN1 | 0.9999 | 0.9999 | 0.0111 | 0.9999 | 0.9999 | 0.0225 |
|  |  | LRNN2 | 0.9999 | 0.9999 | 0.0107 | 0.9999 | 0.9997 | 0.0241 |
|  |  | CFNN1 | 0.9999 | 0.9999 | 0.0040 | 0.9999 | 0.9997 | 0.0260 |
|  |  | CFNN2 | 0.9999 | 0.9999 | 0.0082 | 0.9999 | 0.9999 | 0.0201 |
|  |  | FFNN1 | 0.9999 | 0.9999 | 0.0033 | 0.9999 | 0.9999 | 0.0089 |
|  |  | FFNN2 | 0.9999 | 0.9999 | 0.0041 | 0.9999 | 0.9994 | 0.0101 |
| Wave Height (IN)  (cm) | | ENN1 | 0.9999 | 0.9999 | 0.1380 | 0.9999 | 0.9986 | 0.4014 |
|  |  | ENN2 | 0.9999 | 0.9998 | 0.1234 | 0.9999 | 0.9989 | 0.3224 |
|  |  | LRNN1 | 0.9999 | 0.9993 | 0.0929 | 0.9999 | 0.9949 | 0.2470 |
|  |  | LRNN2 | 0.9999 | 0.9999 | 0.1651 | 0.9999 | 0.9995 | 0.3261 |
|  |  | CFNN1 | 0.9987 | 0.9971 | 0.5728 | 0.9980 | 0.9804 | 1.0543 |
|  |  | CFNN2 | 0.9999 | 0.9999 | 0.0669 | 0.9999 | 0.9984 | 0.4049 |
|  |  | FFNN1 | 0.9999 | 0.9999 | 0.0457 | 0.9999 | 0.9993 | 0.2879 |
|  |  | FFNN2 | 0.9999 | 0.9999 | 0.0760 | 0.9999 | 0.9996 | 0.2258 |
| Wave Height (OUT)  (cm) | | ENN1 | 0.9999 | 0.9999 | 0.0126 | 0.9999 | 0.9999 | 0.0145 |
|  |  | ENN2 | 0.9999 | 0.9999 | 0.0202 | 0.9999 | 0.9999 | 0.0186 |
|  |  | LRNN1 | 0.9999 | 0.9999 | 0.0294 | 0.9999 | 0.9999 | 0.0529 |
|  |  | LRNN2 | 0.9999 | 0.9999 | 0.0321 | 0.9999 | 0.9999 | 0.0622 |
|  |  | CFNN1 | 0.9999 | 0.9999 | 0.0144 | 0.9999 | 0.9999 | 0.0617 |
|  |  | CFNN2 | 0.9999 | 0.9999 | 0.0224 | 0.9999 | 0.9999 | 0.0470 |
|  |  | FFNN1 | 0.9999 | 0.9999 | 0.0339 | 0.9999 | 0.9999 | 0.0623 |
|  |  | FFNN2 | 0.9999 | 0.9999 | 0.0237 | 0.9999 | 0.9999 | 0.0523 |
| Sediment Transport (IN) (cm^3^/s/cm) | | ENN1 | 0.9999 | 0.9999 | 0.0048 | 0.9999 | 0.9999 | 0.0053 |
|  |  | ENN2 | 0.9999 | 0.9999 | 0.0033 | 0.9999 | 0.9999 | 0.0046 |
|  |  | LRNN1 | 0.9998 | 0.9996 | 0.0233 | 0.9999 | 0.9983 | 0.0470 |
|  |  | LRNN2 | 0.9999 | 0.9996 | 0.0169 | 0.9999 | 0.9984 | 0.0332 |
|  |  | CFNN1 | 0.3881 | 0.5107 | 0.5055 | 0.3661 | 0.32833 | 1.3359 |
|  |  | CFNN2 | 0.9997 | 0.9991 | 0.0470 | 0.9999 | 0.9969 | 0.0933 |
|  |  | FFNN1 | 0.9999 | 0.9999 | 0.0157 | 0.9999 | 0.9993 | 0.0411 |
|  |  | FFNN2 | 0.9999 | 0.9999 | 0.0132 | 0.9999 | 0.9996 | 0.0301 |
| Sediment Transport (OUT) (cm^3^/s/cm) | | ENN1 | 0.9999 | 0.9999 | 0.0054 | 0.9999 | 0.9998 | 0.0111 |
|  |  | ENN2 | 0.9999 | 0.9999 | 0.0081 | 0.9999 | 0.9999 | 0.0131 |
|  |  | LRNN1 | 0.9999 | 0.9999 | 0.0111 | 0.9999 | 0.9997 | 0.0219 |
|  |  | LRNN2 | 0.9999 | 0.9998 | 0.0173 | 0.9999 | 0.9989 | 0.0366 |
|  |  | CFNN1 | 0.9997 | 0.9995 | 0.0310 | 0.9994 | 0.9987 | 0.0498 |
|  |  | CFNN2 | 0.9997 | 0.9996 | 0.0319 | 0.9986 | 0.9962 | 0.0907 |
|  |  | FFNN1 | 0.9999 | 0.9999 | 0.0073 | 0.9999 | 0.9998 | 0.0249 |
|  |  | FFNN2 | 0.9999 | 0.9999 | 0.0027 | 0.9999 | 0.9991 | 0.0336 |
|  | **Maximum** | | | | | | | |
| Water Depth (IN)  (m) | | ENN1 | 0.9859 | 0.8020 | 0.9403 | 0.9837 | 0.7941 | 0.8925 |
|  |  | ENN2 | 0.9864 | 0.9514 | 0.7585 | 0.9860 | 0.9469 | 0.7240 |
|  |  | LRNN1 | 0.9869 | 0.8796 | 0.7716 | 0.9862 | 0.9059 | 0.6788 |
|  |  | LRNN2 | 0.9366 | 0.9034 | 0.8284 | 0.9357 | 0.8957 | 0.8312 |
|  |  | CFNN1 | 0.9262 | 0.9075 | 0.8544 | 0.9241 | 0.9102 | 0.7942 |
|  |  | CFNN2 | 0.9408 | 0.8238 | 0.8783 | 0.9350 | 0.8331 | 0.8620 |
|  |  | FFNN1 | 0.9849 | 0.9343 | 0.7815 | 0.9838 | 0.9492 | 0.7378 |
|  |  | FFNN2 | 0.9899 | 0.9063 | 0.8163 | 0.9887 | 0.8711 | 0.8508 |
| Water Depth (OUT)  (m) | | ENN1 | 0.9295 | 0.9065 | 1.0297 | 0.9309 | 0.9113 | 1.0247 |
|  |  | ENN2 | 0.9941 | 0.8432 | 1.5629 | 0.9943 | 0.8456 | 1.5933 |
|  |  | LRNN1 | 0.9067 | 0.8792 | 1.2318 | 0.9097 | 0.8826 | 1.2449 |
|  |  | LRNN2 | 0.9330 | 0.9004 | 1.2383 | 0.9349 | 0.9038 | 1.2006 |
|  |  | CFNN1 | 0.9555 | 0.9154 | 1.1718 | 0.9563 | 0.9171 | 1.1694 |
|  |  | CFNN2 | 0.8863 | 0.9589 | 1.0576 | 0.8898 | 0.9581 | 1.0878 |
|  |  | FFNN1 | 0.9417 | 0.8580 | 1.0697 | 0.9428 | 0.8592 | 1.0716 |
|  |  | FFNN2 | 0.9494 | 0.8966 | 1.1097 | 0.9513 | 0.8999 | 1.0557 |
| Wave Height (IN)  (cm) | | ENN1 | 0.9974 | 0.9717 | 2.6710 | 0.9947 | 0.9669 | 2.7169 |
|  |  | ENN2 | 0.9994 | 0.9621 | 2.6616 | 0.9993 | 0.9445 | 2.0446 |
|  |  | LRNN1 | 0.9941 | 0.9638 | 2.6669 | 0.9916 | 0.8752 | 2.8467 |
|  |  | LRNN2 | 0.9954 | 0.9212 | 3.1328 | 0.9940 | 0.8025 | 3.8398 |
|  |  | CFNN1 | 0.9844 | 0.9104 | 3.5864 | 0.9695 | 0.8517 | 3.7518 |
|  |  | CFNN2 | 0.9895 | 0.9211 | 4.4228 | 0.9777 | 0.9303 | 4.3329 |
|  |  | FFNN1 | 0.9976 | 0.9222 | 4.5097 | 0.9975 | 0.9261 | 2.6770 |
|  |  | FFNN2 | 0.9883 | 0.9678 | 3.0850 | 0.9819 | 0.9574 | 3.3952 |
| Wave Height (OUT)  (cm) | | ENN1 | 0.9942 | 0.9423 | 3.6381 | 0.9934 | 0.9413 | 3.5618 |
|  |  | ENN2 | 0.9998 | 0.9773 | 3.7712 | 0.9998 | 0.9787 | 3.6210 |
|  |  | LRNN1 | 0.9937 | 0.9446 | 3.1526 | 0.9932 | 0.9437 | 3.0076 |
|  |  | LRNN2 | 0.9948 | 0.9850 | 2.6664 | 0.9937 | 0.9857 | 2.4809 |
|  |  | CFNN1 | 0.9979 | 0.9402 | 4.6373 | 0.9976 | 0.9384 | 4.3584 |
|  |  | CFNN2 | 0.9894 | 0.9559 | 5.4044 | 0.9892 | 0.9580 | 4.9741 |
|  |  | FFNN1 | 0.9979 | 0.9430 | 3.7737 | 0.9972 | 0.9298 | 4.1662 |
|  |  | FFNN2 | 0.9894 | 0.9603 | 3.5679 | 0.9881 | 0.9523 | 4.2990 |
| Sediment Transport (IN) (cm^3^/s/cm) | | ENN1 | 0.9996 | 0.9668 | 4.9588 | 0.9983 | 0.5355 | 3.2415 |
|  |  | ENN2 | 0.9989 | 0.8778 | 5.9744 | 0.9953 | 0.9764 | 3.0995 |
|  |  | LRNN1 | 0.9983 | 0.9953 | 3.4794 | 0.9954 | 0.9536 | 3.3873 |
|  |  | LRNN2 | 0.9953 | 0.9747 | 4.5166 | 0.9691 | 0.9504 | 4.2983 |
|  |  | CFNN1 | 0.9980 | 0.7658 | 6.2670 | 0.9847 | 0.6266 | 6.0368 |
|  |  | CFNN2 | 0.9975 | 0.9886 | 3.9103 | 0.9844 | 0.7117 | 6.1496 |
|  |  | FFNN1 | 0.9983 | 0.9927 | 4.7836 | 0.9918 | 0.9815 | 3.8296 |
|  |  | FFNN2 | 0.9822 | 0.9482 | 9.5585 | 0.9350 | 0.9597 | 6.4523 |
| Sediment Transport (OUT) (cm^3^/s/cm) | | ENN1 | 0.9961 | 0.9915 | 11.1589 | 0.9543 | 0.8036 | 9.1024 |
|  |  | ENN2 | 0.9997 | 0.9881 | 13.0220 | 0.9886 | 0.5732 | 10.8376 |
|  |  | LRNN1 | 0.9823 | 0.9215 | 17.8579 | 0.7470 | 0.8917 | 10.8105 |
|  |  | LRNN2 | 0.9961 | 0.9906 | 11.5271 | 0.9379 | 0.8684 | 9.7350 |
|  |  | CFNN1 | 0.9613 | 0.9792 | 22.7864 | 0.7014 | 0.6422 | 15.9658 |
|  |  | CFNN2 | 0.9812 | 0.9565 | 14.5655 | 0.7438 | 0.6715 | 13.7036 |
|  |  | FFNN1 | 0.9968 | 0.9289 | 21.6972 | 0.9582 | 0.7015 | 12.9478 |
|  |  | FFNN2 | 0.9968 | 0.9867 | 9.6717 | 0.9552 | 0.8886 | 9.1276 |
