# Supplementary figures and images for: A novel framework for the evaluation of coastal protection schemes through integration of numerical modelling and artificial intelligence into the Sand Engine App
Source: Sci Rep. 2023 May 27;13:8610. doi: 10.1038/s41598-023-35801-5 (PMC10224936; doi:10.1038/s41598-023-35801-5)

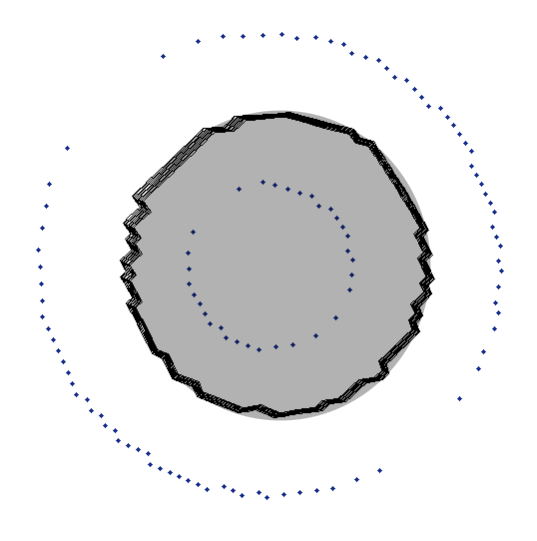


Figure S1. Sand engine (shaded area) of radius 2 km with inner and outer observation points (blue dots)

Supplement: Supplementary file 2 — Supplementary Figure S1. [file 41598_2023_35801_MOESM2_ESM.docx]
